# Randomized controlled trial protocol to improve multisensory neural processing, language and motor outcomes in preterm infants

**DOI:** 10.1186/s12887-019-1455-1

**Published:** 2019-03-19

**Authors:** Mary Lauren Neel, Paul Yoder, Pawel J. Matusz, Micah M. Murray, Ashley Miller, Stephanie Burkhardt, Lelia Emery, Kaleigh Hague, Caitlin Pennington, Jessica Purnell, Megan Lightfoot, Nathalie L. Maitre

**Affiliations:** 1 Nationwide Children’s Hospital Division of Neonatology & Center for Perinatal Research, The Ohio State University, 5th floor; 575 Children’s Crossroads, Columbus, OH 43215 USA; 2 Department of Special Education, Vanderbilt University, Nashville, TN USA; 3 Information Systems Institute at the University of Applied Sciences Western Switzerland (HES-SO Valais), 3960 Sierre, Switzerland; 4 The LINE (Laboratory for Investigative Neurophysiology), Department of Radiology and Clinical Neurosciences, University Hospital Center and University of Lausanne, Lausanne, Switzerland; 5 Electroencephalography Brain Mapping Core, Center for Biomedical Imaging (CIBM) of Lausanne and Geneva, Lausanne, Switzerland; 6 Department of Ophthalmology, Fondation Asile des Aveugles, Lausanne, Switzerland; 7 Department of Hearing and Speech Sciences, Vanderbilt University, Nashville, TN USA

**Keywords:** Multisensory, Intervention, Preterm, Neurodevelopment, Event-related potential, Sensory function

## Abstract

**Background:**

Premature infants are at risk for abnormal sensory development due to brain immaturity at birth and atypical early sensory experiences in the Neonatal Intensive Care Unit. This altered sensory development can have downstream effects on other more complex developmental processes. There are currently no interventions that address rehabilitation of sensory function in the neonatal period.

**Methods:**

This study is a randomized controlled trial of preterm infants enrolled at 32–36 weeks postmenstrual age to either standard care or standard care plus multisensory intervention in order to study the effect of multisensory intervention as compared to standard care alone. The study population will consist of 100 preterm infants in each group (total *n* = 200). Both groups will receive standard care, consisting of non-contingent recorded parent’s voice and skin-to-skin by parent. The multisensory group will also receive contemporaneous holding and light pressure containment for tactile stimulation, playing of the mother’s voice contingent on the infant’s pacifier sucking for auditory stimulation, exposure to a parent-scented cloth for olfactory stimulation, and exposure to carefully regulated therapist breathing that is mindful and responsive to the child’s condition for vestibular stimulation. The primary outcome is a brain-based measure of multisensory processing, measured using time locked-EEG. Secondary outcomes include sensory adaptation, tactile processing, speech sound differentiation, motor and language function, measured at one and two years corrected gestational age.

**Discussion:**

This is the first randomized controlled trial of a multisensory intervention using brain-based measurements in order to explain the causal effects of the multisensory intervention on neural processing changes to mediate neurodevelopmental outcomes in former preterm infants. In addition to contributing a critical link in our understanding of these processes, the protocolized multisensory intervention in this study is therapist administered, parent supported and leverages simple technology. Thus, this multisensory intervention has the potential to be widely implemented in various NICU settings, with the opportunity to potentially improve neurodevelopment of premature infants.

**Trial registration:**

NIH Clinical Trials (clinicaltrials.gov): NCT03232931. Registered July 2017.

## Background

Every year, half a million infants are born prematurely in the United States and 15 million worldwide [1, 2]. The vast majority of preterm infants will have only moderate to mild impairments or delays in early childhood, with intellectual and behavioral consequences of prematurity only apparent at school age and beyond [3–7]. Almost all preterm infants suffer from atypical brain maturation and its developmental consequences resulting from interactions between brain immaturity and premature extra-uterine sensory experience [8–15]. Brain development in the neonatal period is experience-dependent, yet the neonatal intensive care experience is largely comprised of atypical sensory stimuli [11, 13, 15–22]. The critical importance of establishing functional sensory systems in infancy as the basis for all higher order processes (cognition, communication, behavioral adaptation) has been demonstrated in both animal models and humans [13, 15, 23–25]. Preterm infants at discharge to home often have altered sensory reactivity and modulation in response to their environment, which are associated with negative neurodevelopmental outcomes in childhood [20, 22, 26].

Parents are essential in scaffolding early learning and development, especially with regards to early sensory exposures and responses [27–29]. In particular, parental linguistic input is a key concept in learning language [9, 30–36]. Importantly, this input is more effective when it is contingent and immediate (i.e., language is presented immediately after and only upon infant action) [9, 30–36]. This precept holds true even in early pre-linguistic phases, when infants differentiate among speech sounds, which is necessary for later development of higher-order language milestones [9, 30–36]. Another type of scaffolding provided by parents is the multisensory support of skin-to-skin care (STS) contact, which helps maintain quiet and well-regulated states in immature infants who have frequent autonomic instability [37–40]. Unfortunately, providing STS is often challenging for parents who often have to travel long distances to see their infant, while balancing responsibilities of other children and jobs, in addition to potential unreliable transportation and/or lack of social support [41–45].

Multisensory processes (MSPs) are rarely studied in neonates, yet in children and adults MSPs are essential to building a coherent and unified perception of the world, a foundation for learning and social interactions [46]. There are currently no mechanistically proven interventions that address rehabilitation of sensory function in the neonatal period, when brain-plasticity is at its greatest and when improvements can have an exponential downstream effect on later neurodevelopment [47–50]. The few associative studies of sensory processing, while critical, have not examined causal effects of interventions on neural processing changes to mediate neurodevelopmental outcomes. More infant-directed speech is associated with better language outcomes in the first 18 months and increased STS care with improved autonomic system stability and muscle tone in the hospital, and improved behavioral and motor outcomes in infancy [30–33, 37, 51–53]. The current study is the first to use brain-based measures to test predictions regarding how the brain changes in response to multisensory treatment, which in turn affects functional outcomes.

To accomplish our goals, we designed a prospective, interventional Randomized Controlled Trial (RCT) in preterm infants aimed at restoring more typical multisensory, rather than unisensory, processing. Our multisensory intervention using parents’ voice and nurturing touch can be administered regularly in the NICU (Neonatal Intensive Care Unit) during sensitive periods of sensory development, even when parents cannot always be present. Our test of this multisensory intervention will involve sessions of standardized, therapist-administered, multisensory stimulation. This treatment will combine contingent presentation of the recorded mother’s voice delivered using a suck-activated system during holding with supportive vestibular stimulation and tactile containment against a cloth scented by parent contact on the therapist’s chest. This treatment will be tested in an internally-valid RCT. In addition to testing the efficacy of the treatment on gold-standard measures of language and motor functioning, we will also test whether the treatment effect is due to intermediate treatment effects on multisensory and unisensory processing. Understanding the mechanism by which the treatment works is important for laying the foundations of future improvements and potential recommendations for treatment that might be useful for widespread use even in lower resource settings with the ultimate goal of improving neurodevelopmental outcomes for these premature infants.

Our research aims are as follows:

1. To demonstrate that preterm infants receiving a standardized, parent-supported, auditory-tactile-olfactory-vestibular intervention in addition to standard of care in the NICU will have more typical cortical multisensory processing at discharge and better sensory adaptation and motor and language outcomes than infants receiving only standard of care.
2. To test the role of multisensory responses at discharge in mediating intervention effects on later sensory adaptation, and motor and language outcomes.
3. To explore the role of unisensory responses in mediating intervention effects on later motor and language outcomes.

## Methods

### Study design

**Fig. 1:**
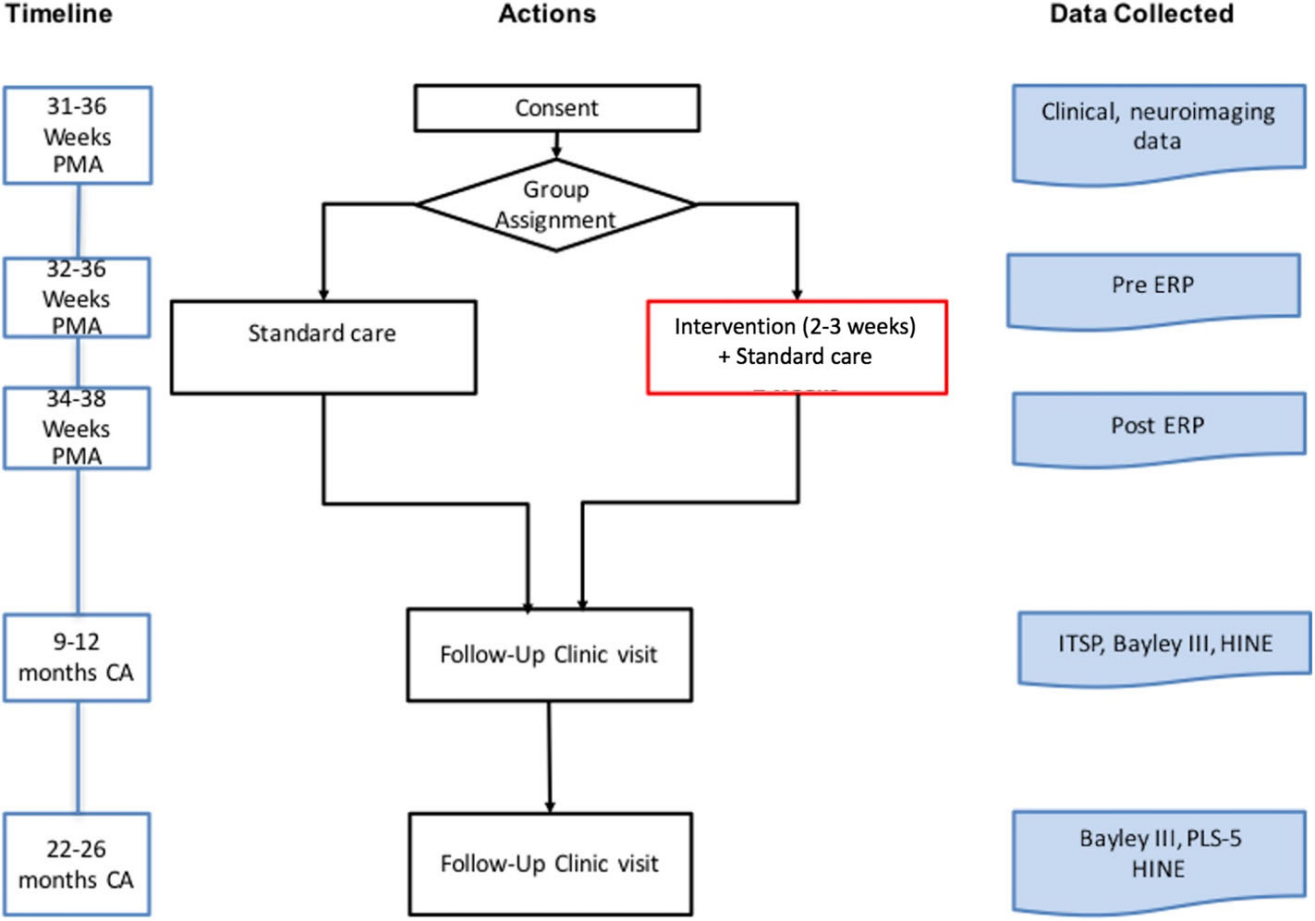
Study process flow. (Bayley III, Bayley Scales of Infant and Toddler Development Third ed.; CA, corrected age; ERP, Event-related Potential measurement; HINE, Hammersmith Infant Neurological Exam; ITSP, Infant Toddler Sensory Profile; PLS-5, Preschool Language Scales-5; PMA, post menstrual age)

In order to test these aims, we will conduct an RCT of a multisensory (MS) intervention with 200 hospitalized preterm infants in our Level IV NICU (Fig. 1). Enrollment is planned from October 2018–October 2020. For infants who meet inclusion criteria at 31 weeks, parental consent will be obtained, parent’s voice recorded and parent-scented clothes collected. Infants will then be randomized to the control or intervention group. Both control and intervention groups will receive standard care, which includes STS care on parent, when present, and playing of parents’ voice non-contingent on infant suck. Infants assigned to the MS group will also receive 20 sessions of the standardized MS intervention over 2–3 weeks, starting at 32 0/7 weeks postmenstrual age (PMA) or at enrollment prior to 36 weeks. ERP (Event-related potential) testing will be performed prior to the intervention and at discharge, which occurs at 36 weeks PMA on average but later in the most preterm infants. All infants will be seen at the NICU Follow-Up Program clinics at 9–12 months PMA (Year 1) and 22–24 months PMA (Year 2), when Bayley III and Hammersmith Infant Neurological Exam are performed per standard care. At the Year 1 visit, the research coordinator administers the Infant Toddler Sensory Profile (ITSP), and at the Year 2 time-point, the research team administers the PLS-5 (Preschool Language Scales-5) [54].

### Study participants

The study population will comprise 200 preterm infants. All races and ethnicities will be included. All materials will be translated by an interpreter for non-English speaking participants.

### Inclusion and exclusion criteria

Inclusion criteria are PMA 32 0/7 weeks gestation - 36 0/7 weeks gestation. Exclusion criteria are ventilation using an endotracheal tube, major congenital malformations, family history of genetic hearing loss, and use of sedatives or seizure medications. Major congenital malformations may prevent accurate ERP measurements, and the medications above may mask sensory processing as assessed by ERPs.

### Randomization and RCT design elements

Assignment to groups will be carried out using unified reproducible methods with a permuted block randomization scheme with random block size. Allocation will be concealed from all study personnel who could influence scores [55]. Except for parent reports, examiners will be blind to treatment assignment. Recordings of parents’ voices will be obtained from both groups with the intent of masking parents to assignment.

#### Adherence to treatment protocol

A highly manualized protocol for the MS treatment is monitored and 10% of videos are reviewed by an independent reviewer. Standard care is also monitored using nursing logs in both groups. These measures should help ensure uniform, high-quality implementation of the MS treatment. Sufficiently and randomly sampled fidelity of treatment (FOT) measures will be collected [56]. Treatment is provided in the NICU by experienced therapists.

#### Total attrition

Attrition is expected to be less than 10% [25, 57–59]. Should motion artifacts occur, we will retest patients with insufficient data within 24 h. For the Bayley III, should children prove unable to complete follow-up testing during scheduled visits, they will be rescheduled within two weeks of the scheduled visit. Testers are trained to perform the Bayley III in the home environment, if necessary. The ITSP can be performed over the phone or through the mail if necessary and will be performed within one week of a missed visit. Analysis will follow an intent-to-treat protocol [60]. As such, all randomized participants will be analyzed and missing data will be handled using multiple imputation [61].

#### Differential attrition

Differential attrition is unlikely because children must be in the NICU during the treatment phase due to health concerns. There are no non-NICU treatments during the treatment phase. Thus, compensatory responses due to parental displeasure of group assignment are unlikely.

#### Covariates

Biological variables are factored into our research design as potential covariates. These include gestational age at birth, sex, presence of severe white neural injury on neuroimaging (intraventricular hemorrhage grade III or IV, periventricular leukomalacia, cerebellar hemorrhage, ischemic or thrombotic injury), presence of systemic inflammatory conditions (history of necrotizing enterocolitis Bells stage IIA or above, culture documented sepsis or meningitis, moderate or severe bronchopulmonary dysplasia per modified Shennan definition), cumulative caffeine exposure post intervention, total parental STS time during the study period, and pretreatment status on the General Movements Assessment exam. Several pretreatment covariates will be statistically controlled if needed. These variables will be quantified at the pretreatment period, as past research or theory suggests they may be associated with neurodevelopmental outcomes or sensory processing [58, 62–65]. If preliminary tests of between-group differences of these pretreatment variables and the pretreatment variables showing between-group differences are associated with putative mediators or neurodevelopmental outcomes, these will be statistically controlled. Their statistical interaction with treatment group must be tested as part of the process of determining whether they should be statistically controlled. Thus, the possibility that treatment varies as a function of these biological variables will also be explored.

## Intervention design

### Standard care

The standard care of infants currently follows two medical protocols; one for STS holding and one for exposure to the parent’s voice. The protocols will be monitored as follows:

#### Exposure to recorded parent’s voice

Preterm infants in the NICU currently receive non-contingent recordings of the parent’s voice during two to three 20-min sessions daily. Recordings are standardized [66]. Recordings are then played through a sterilizable music player with a median volume of 45 dB and a maximum volume of 55 dB [67]. Monitoring of compliance with standard care will be accomplished through daily review of the medical record to determine the number of times the recording is played and to ensure that the recorded voice is never played at the same time as STS.

#### Skin-to-skin holding

Per standard care, STS care will be carried out by parents in both groups. Parents in the NICU currently use either their hands or positioners to facilitate prolonged STS. Infants are placed in a prone position with head positioned over the sternum, allowing transmission of breath and heart sounds to the developing ear. Deeper pressure is applied to offer support and feedback to the child’s bottom. When primary caregivers are not comfortable with direct STS contact (e.g. are not a direct relation to the child, or parent or child skin problem), a thin single-use hospital gown that is not previously imprinted with the parent’s scent is used to facilitate the experience without hindering sound transmission. In order to ensure safety during STS holding, vital signs including heart rate, breathing patterns and rate, oxygen saturation and temperature are continuously and automatically monitored with preset alarms per unit protocol. If any negative deviation from the infant’s daily vital sign patterns occurs, the nurses examine the infant and decide whether to stop the STS. To monitor STS, we will review the medical record for daily start time, duration, and caregiver during STS. We will also record any deviations from autonomic stability during STS (tachy/bradycardic events, tachypnea, or apnea).

### Multisensory intervention

The MS intervention will be carried out in addition to standard care and will include the following components: holding and light pressure containment of the infant against the hospital-gown covered chest of the therapist for tactile and non-specific auditory stimulation simultaneous with playing of the mother’s voice contingent on infant pacifier sucking for the first 20 min of holding [68]. Additionally, a cotton square from a T-shirt scented with parent’s skin will be placed under the infant’s face on the therapists’ chest, to provide olfactory stimulation without risk of suffocation. The final component of the MS intervention is the carefully regulated, mindful breathing of the therapist for infant vestibular stimulation. The 20 intervention sessions will be dispersed across 2–3 weeks. On the occasion that the parent is present, parental STS will always take precedence over the intervention and the intervention session will be separated from previous parental holding by a minimum of 2 nursing care intervals (6 h) in one 24-h period. Infant autonomic stability and negative deviations during the MS intervention with the therapist will be recorded as above for parent STS care.

#### Contingent parent’s voice exposure

We will use the Pacifier Activated Lullaby® (PAL®) device, a 510 k FDA approved digital music delivery system that integrates a sensor, a pacifier routinely used in the NICU, and a receiver [68]. It delivers a predetermined 10 s of recorded parent’s voice singing lullabies upon detection of a suck that meets a preset pressure threshold. The original systems were modified for research use by decreasing the lower limit of activation thresholds for delivering the recording [69]. Minimal effort is required to trigger the device. However, the settings ensure that regular attempts are needed to continue to receive continual presentation of the recording of mother’s voice by requiring another suck after 10 s. The auditory simulation with PAL will be provided when the infants are still awake (i.e., during the first 20 min of holding).

#### STS holding

The therapist will wear a clean single use cotton hospital gown and wrap the “kangaroo” positioner securely over the top. The positioner will allow containment and even deep pressure and PAL operations. Should assistance be required, personnel will use the unit standard personal voice-activated call system to request a team member and minimize disruptions to the infant. The scented cotton placed next to the infant’s nose will be obtained by having the parent wear for a timed interval, cutting it with gloved hands and storing it in a sealed bag immediately upon removal. Cloth will be replaced if contaminated with infant’s bodily fluids.

#### Relaxation training of the MS therapy team

Because effects of STS holding are thought to be partly mediated by the holder’s heart and respiratory rate and because therapists are providing vestibular stimulation to infants, it is essential to maintain calm throughout the PAL administration in the way that parents would while holding their infant without additional activities. To promote this, therapists will attend a workshop on mindfulness techniques and practice this prior to start of the intervention [70]. Data on therapist heart and respiratory regulation in simulated stressful sessions will be obtained before, after, and one month after consistent training to ensure proficiency and fidelity before start of treatment. During intervention MS sessions, therapists will wear Spire Stone (Spire Inc., San Francisco CA) breathing rate monitors which provides a gentle reminder should they need return to a calmer state.

### Ensuring high intervention fidelity

A script for the therapy session detailing the essential steps and sequence of the procedure is produced in a video with a checklist of critical elements [71]. All study therapists and an independent observer in the laboratory study the videos prior to beginning implementation. During a common training phase, the observer scores all therapists on all steps using a Likert-scaled rating system. The therapists also score themselves on the fidelity rating scale in order to immediately compare their self-assessments with those of the observer. The training phase is concluded when there is 90% adherence to the protocol and concordance between therapists and observer scoring. A random sample of 10% of all MS treatment sessions for each patient and intervention therapist will be assessed for fidelity with the checklist rating scale by the trained observer.

### Outcomes assessment methods

Our primary outcome is multisensory response and our secondary outcome is neurodevelopmental outcomes, including sensory adaptation, motor, language, tactile processing, and speech sound differentiation (Table 1).

**Table 1:** Constructs and procedures

Construct	Procedure(s)	Timing
Multisensory response (Auditory-Tactile processing)	ERP to simultaneous puff + speech sound	Pre- and post-intervention (near discharge)
Sensory functioning	ITSP	9–12 months
Language	Bayley III PLS-5	22–26 months
Motor	Bayley III	9–12 months 22–26 months
Tactile processing Functional tactile connectivity	ERP to calibrated air puff ERP to calibrated air puff	Pre/Post intervention Pre/Post intervention
Speech Sound differentiation	ERP to 6 speech sounds	Pre/Post intervention

This table shows the experimental constructs, procedures, and timing of variables for analysis. (*ERP* Event-Related Potential, *ITSP* Infant Toddler Sensory Profile, *PLS-5* Preschool Language Scales-5)

### Sensory processing measurement by ERP

#### ERP recording

A high-density array of 128 electrodes embedded in soft sponges (Hydrocel Sensor Net, EGI, Inc., Eugene, OR) will be used to record ERPs with a sampling rate of 1000 Hz, filters set to 0.1–400 Hz. Recording of brainwaves will be controlled by Net Station (v. 4.3; EGI, Inc., Eugene, OR). E-Prime (v. 4.0, PST, Inc., Pittsburgh, PA) software will control stimulus delivery.

#### Stimulus-presentation paradigms

The ERP procedure involves blocks of trials from four conditions: multisensory (simultaneous speech sound-puff), puff alone, speech sound alone, and sham puff in a randomly generated sequence. To prevent habituation, no more than 2 repetitions of a condition occur in a row, with inter-trial intervals varying randomly between 2000 and 2500 ms. The “light touch” stimulus is an air puff emanating from a nozzle positioned above the skin of the palmar surface of the right hand secured in a mold holder. Another mold holder connected to the second nozzle is placed 15 cm away at midline (sham condition). The entire test session generates 60 trials per condition and lasts 8–10 min [72, 73]. The speech sound condition is a computer-generated woman’s voicing of one of six syllables (i.e. /ba/, /da/, /ga/, /bu./, /du/, /gu/) delivered in a free field setting using a microphone placed at midline 15 cm. The speech stimuli are computer-synthesized consonant-vowel syllables as previously published [74]. The syllables are presented at 65 dB SPL(A) (sound pressure level). More than the 2 key stimuli (/bu./ and /gu/) are presented to prevent habituation [59].

#### Preparation and analysis of ERP data

The recorded data will be filtered using a 0.3–40 Hz bandpass filter and segmented on stimulus onset to include a 200-ms pre-stimulus baseline and a 500-ms post-stimulus interval. Electrodes will be referred to Cz and re-referenced offline to an average reference. Resulting segments will be screened for motor/ocular artifacts using standard algorithms in NetStation, followed by a manual review. We will utilize previously published time windows and electrode clusters.

#### Index of multisensory processing (IMP)

We have already identified template maps over the cumulative 500 ms post-stimulus time window using a topographic cluster analysis (i.e., Topographic Atomize & Agglomerate Hierarchical Clustering approach) for full-term (FT) and preterm (PT) infants, which account for 96.3% of the global variance in ERP response to the MS stimulus [75]. Using a within-participant spatial correlation process (i.e., cases are electrodes and the two variables are (a) participant’s ERP and (b) template map value), we can identify which template map (FT or PT) best fits each time-samples’ observed topographical data for each participant. From this procedure, we calculate the percentage of time the topographical pattern of the participant’s ERP to MS stimuli is most like the FT template map. We call this index the IMP. This index is expressed as a percentage from 100% (all time samples show nearly-typical activation) to 0% (no time samples show nearly-typical activation).

#### *Infant Toddler Sensory Profile (ITSP*) [76–78]

The ITSP is the most validated test for the behavioral evaluation of sensory processing. This parent-rated questionnaire has 48 questions, addressing five sensory processing sections and a General Measure. One variable from this instrument is the infant’s neurological threshold (tendency to respond to sensory stimuli). Raw individual section scores are also provided. The ITSP has been used in large studies of preterm infants.

#### *Bayley Scales of Infant and Toddler Development (Bayley III) — 3rd Edition* [79]

The Bayley III is the gold standard for the evaluation of former NICU graduates, especially preterm infants. We will use the language and motor composite standard scores for corrected age. The Bayley is currently administered in the Follow-Up Clinic by trained examiners who undergo yearly retraining by a Gold-standard examiner.

### Analysis plan

#### Power/sample size

A total sample size of 200 (100 in each group) will be recruited. Although attrition in past similar work has been much lower, we estimate power under an assumption of 10% attrition (i.e., 180). Because pilot studies afford effect size estimates with wide confidence intervals due to small sample sizes, power analysis results were conducted for the effect size available from pilot data and lowest effect size that the after-attrition sample size affords. Feasibility of the expected effect size is then evaluated. Power analyses are based on primary variables (multi- and uni- sensory processing). If between-group differences occur on the putative covariates (i.e., pretreatment variables related to the outcomes) and they are associated with putative mediators or outcomes, the pretreatment variables will be statistically controlled after ensuring that the homogeneity of slopes assumption has been met. Pretreatment ERP variables will be statistically controlled regardless of the effect size of between-group differences to improve effect size estimates. Missing data will be handled using multiple imputation [61].

### Statistical analysis

Our first aim is to demonstrate that preterm infants receiving a standardized multisensory auditory-tactile-olfactory-vestibular intervention in addition to standard of care in the NICU will have more typical cortical multisensory processing at discharge and better sensory adaptation and motor and language outcomes than infants receiving only standard of care. For Aim 1, the between-group differences will be tested on ERP-measured multisensory processing at discharge from the NICU, sensory reactivity and adaption at Year 1, and motor and language ability at Year 2. When the dependent variable is continuous, we will use ANCOVAs when controlling for pretreatment variables and independent sample t-tests when there is no need for a covariate. When testing the nominal form of the sensory reactivity and adaptation outcome, we will use logistic regression.

### Statistical power

Using PASS software, the estimated power using a 1-tailed test for an effect size based on pilot data with a sample size of 180 is over 99%. Even if the actual effect size is *g = .35,* we will have over 80% power to detect the effect under the proposed conditions*.*

Our second aim is to test the role of multisensory responses at discharge in mediating intervention effects on later sensory adaptation and motor and language outcomes.

**Fig. 2:**
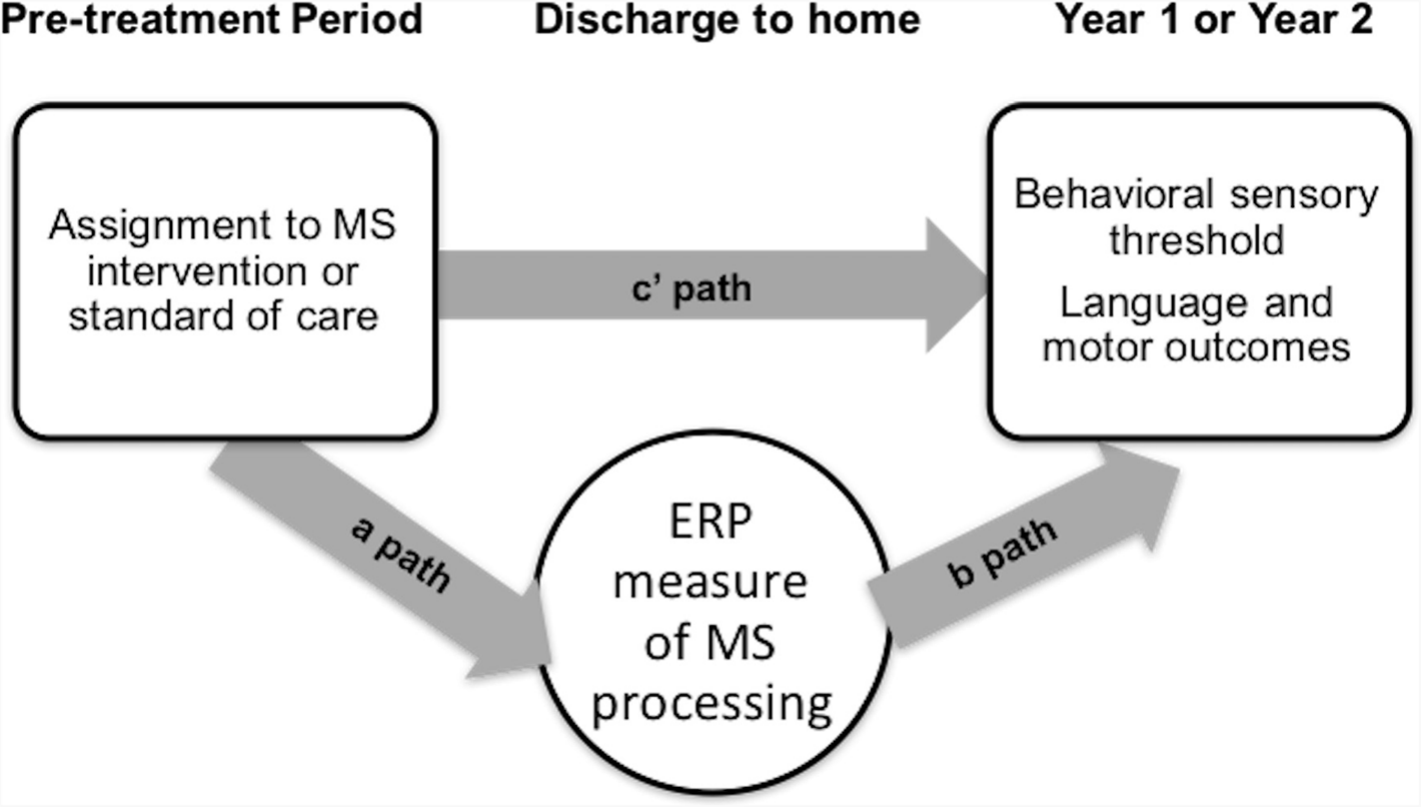
Mediation analysis model. The a-path is the main effect of treatment on the ERP measure of multisensory processing. The b-path is the association of the ERP measure of multisensory processing with the outcome controlling for the treatment group. The indirect effect (a-path coefficient * b-path coefficient) will be tested for significance in this study. (*ERP, Event related potential; MS, multisensory*)

Mediation models are presented in Fig. 2. The indirect effect (the product of the a-path coefficient * the b path coefficient) will be tested for significance using the bias-corrected bootstrap method [80]. The a-path is the main effect of treatment on the ERP measure of multisensory processing. The b-path is the association of the ERP measure of multisensory processing with the outcome controlling for the treatment group.

### Statistical power

Using simulation data, an indirect effect produced by paths each with at least the mid-point between small and medium effect sizes (0.26; i.e., at least 6.8% of the variance of the criterion variable accounted for in each path) will be detected with over 80% power when using a sample size of 180 [81].

Our third aim is to explore the role of unisensory responses in mediating intervention effects on later motor and language outcomes*.* The putative mediators are the two unisensory processing (i.e., speech processing or touch) variables. The putative mediators are speech processing for the language outcome and touch processing for the motor outcome.

### Statistical power

With regards to the a-path, the sample-size-adjusted standardized mean difference on speech processing effect size from the contingent parent-voice versus control (a treatment that is a subset of the proposed treatment) contrast was large. The proposed treatment will provide more nurturing touch than infants in control group and nurturing touch has been associated with better touch perception experience in our preliminary studies.

### Data management

All data will be stored using Research Electronic Data Capture (REDCap), a secure, web-based application designed to support data capture for research studies by building and managing online surveys and databases [82]. Support for REDCap is available through Nationwide Children’s Hospital.

### Ethics

This study is based on our previous work and the NIH call for applications regarding multisensory processing and interventions [83]. This NIH-funded study was approved by the Nationwide Children’s Hospital IRB. It was assigned a risk level 1 (no greater than minimal risk). Informed consent will be obtained from the parent/guardian in accordance with the IRB protocol. A data safety monitoring committee consisting of the principal investigator, study coordinator, NICU nursing clinical leader, clinical program manager of developmental therapists in the NICU, and a parent of a NICU graduate on the parent advisory committee will further oversee the ongoing study.

## Discussion

Previous studies prove that early sensory experiences shape brain development in former preterm infants [13, 15, 23, 24]. A few associative studies have demonstrated improved neurodevelopmental outcomes with supportive, targeted sensory input (ie STS or infant directed speech) [19, 30–33, 51–53]. A few other studies have examined associations between protocolized multisensory interventions, such as Auditory, Tactile, Visual and Vestibular Stimulus (ATVV) with short term NICU outcomes, such as feeding, behavioral states, and neuromotor assessments [84–87]. Only one study of ATVV with 37 preterm patients examined neurodevelopment at one year [88], and no studies have examined neurodevelopment after one year. To our knowledge, ours is the first RCT to design and test a protocolized MS intervention using brain-based measurements in order to elucidate the causal effects of the MS intervention on neural processing changes to mediate neurodevelopmental outcomes. This study provides a critical link in further understanding interactions between brain development, plasticity, environmental input, and subsequent neurodevelopment in this particularly vulnerable population of infants, and offers the possibility of an intervention that could be implemented in a variety of NICU settings.

## Abbreviations

ERP: Event-related potential
FOT: Fidelity of treatment
FT: Full-term
IMP: Index of multisensory processing
IRB: Institutional Review Board
ITSP: Infant Toddler Sensory Profile
MS: Multisensory
MSP: Multisensory processes
NICU: Neonatal Intensive Care Unit
PAL®: Pacifier Activated Lullaby®
PLS-5: Preschool Language Scales-5
PMA: Postmenstrual age
PT: Preterm
RCT: Randomized Controlled Trial
REDCap: Research Electronic Data Capture
SPL: Sound pressure level
STS: Skin-to-skin care
